# P27 Fever, falling counts and hyperferritinaemia in a septic patient - could it be haemophagocytic lymphohistocytosis?

**DOI:** 10.1093/rap/rkae117.058

**Published:** 2024-11-05

**Authors:** Urwah Ahmed, Sadaf Saeed, Jessica Gunn, Iman Ali, Alexis Jones, Jessica Manson

**Affiliations:** Cheltenham General Hospital, Cheltenham, United Kingdom; Gloucester Royal Hospital, Gloucester, United Kingdom; Cheltenham General Hospital, Cheltenham, United Kingdom; Cheltenham General Hospital, Cheltenham, United Kingdom; University College London Hospital, London, United Kingdom; University College London Hospital, London, United Kingdom

## Abstract

**Introduction:**

Haemophagocytic lymphohistocytosis (HLH) is an under-recognised hyperinflammatory syndrome, and a life-threatening infection mimic. It has an overall one-year survival of only 56% and must be actively sought out in any febrile, unwell patient with cytopenias. Management requires controlling the cytokine storm in parallel with identifying the underlying trigger. Infection can both be a trigger, and a treatment complication of the potent immunosuppression required for HLH management.

I present the case of refractory HLH due to an unknown trigger, requiring etoposide to turn off the cytokine storm, only to result in mortality due to insurmountable superadded infections despite full antimicrobial prophylaxis.

**Case description:**

A 33-year-old previously well female presented with a week’s history of generalised rash, fevers, malaise and polyarthralgia. Although no infective cause was identified despite an extensive search, empirical antibacterial treatment was commenced as she appeared septic with elevated inflammatory markers. Other investigations demonstrated deranged liver function tests (LFTs), ferritin resulted as > 1500ug/L, splenomegaly and reactive lymphadenopathy (but no malignancy) on imaging. Management for adult-onset Still’s disease was initiated in parallel to investigations to exclude other diseases and enable calculation of an HScore.

Treatment was escalated to IV methylprednisolone and anakinra due to clinical and biochemical deterioration (ferritin >13000ug/L). She was admitted to the intensive care unit (ICU) for observation as a high mortality risk was identified early. Anakinra was briefly paused as worsening LFTs were thought to be drug- induced but resumed on recurrence of fever, rash and increasing HScore. Bone marrow biopsy demonstrated haemophagocytes, but no malignancy.

Local and specialist centre MDTs were involved throughout. The University College London Hospital (UCLH) HLH service advised adding IVIG and antimicrobial prophylaxis. Further deterioration with falling blood counts, rising lactate and cardiogenic shock necessitated intubation. The consensus MDT view was to give etoposide for cardiac dysfunction. The local ICU team referred her for extracorporeal membrane oxygenation (ECMO) but were advised that she was not an appropriate candidate.

Initial dramatic improvement with etoposide (reflected in decreasing lactate and organ support required) was followed swiftly by profound cytopenias and rising CRP. *Candida glabrata* was isolated from line cultures, and imaging demonstrated evidence of paracolitis, thought to be CMV colitis. She was appropriately treated for both and transferred to UCLH for ongoing care. She developed a further insurmountable invasive fungal infection of her palate and bowel (both requiring surgical intervention). The latter led to an acute abdominal bleed, ultimately being the cause of her death.

**Discussion:**

This case highlights the importance of early consideration of HLH as a mimicker of severe sepsis and demonstrates its diagnostic principles, management of standard and refractory disease, as well as complications of the potent immunosuppressive treatment utilised.

Even when the diagnosis is reached, system limitations may hinder the delivery of optimal care in settings where HLH is less often encountered and formal management pathways may be lacking. For example, our laboratory did not initially process daily ferritins and provided results only in large increment values until we discussed the importance of these in assessing treatment response. This highlights the need to improve education amongst laboratory colleagues. Additionally, there may be a lack of clarity regarding the parent team for these patients, particularly if there is no out-of-hours rheumatology service.

Discussions around the use of etoposide (third line treatment in refractory HLH) and ECMO were particularly interesting. While the local team kept the ULCH MDT and patient’s next of kin fully informed, the patient was intubated and ventilated and thus not able to discuss the potential serious long-term sequelae of etoposide (including secondary neoplasms and fertility impact). Given the nature of etoposide as a ‘last resort’ drug, this is perhaps a theoretical issue rather than a practical one but all prescribers should be aware. The decision that she was not an appropriate candidate for ECMO was agreed on by three ECMO centres. This was due to the high risk of cannulation due to thrombocytopenia, poor prognostic factors, hyperlactatemia despite multiorgan support and established cardiogenic shock. Any improvement with etoposide would likely take days and thereby increase the complications of ECMO. This showcases the importance of not shying away from seeking multiple expert opinions when making tough decisions and thinking rationally about treatment escalation limitations even in very emotionally charged scenarios.

**Key learning points:**

• Consideration of HLH as a sepsis mimic, particularly in the presence of the 3Fs of fever, falling counts and hyperferritinaemia, is critical. Treatment should be initiated in parallel to identifying an underlying trigger, recognising also that these patients deteriorate quickly and should be cared for in an ICU setting.

• Patients with HLH have an increased susceptibility to severe opportunistic infections. This can be due to the underlying trigger for HLH, or cytopenias which can be secondary to HLH itself or the potent immunosuppressives required in its management. Here, etoposide switched off the refractory cytokine storm (as evidenced by the patient initially surviving a lactate >20 mmol/L) but did contribute to serious myelosuppression. Therefore, comprehensive antimicrobial prophylaxis and treatment of superadded infections are key tenets in HLH management.

• Whilst recognising iatrogenic complications, it is also critical to remember that HLH itself is a multiorgan disease. Here, worsening LFTs and cardiac function may represent HLH disease activity rather than being drug induced. Whilst in hindsight one could argue that earlier recognition of this may have resulted in treatment escalation rather than brief cessation of anakinra, this decision-making process is much more opaque in the context of only a presumptive working diagnosis with few ‘positive’ results but a clear deterioration in a parameter on initiating a new drug.

• MDT working is required at all stages of HLH management; where necessary, regional and national expertise should be utilised. The UCLH HLH MDT represents a prototype of key specialties required: rheumatology, haematology, intensive care and infectious diseases. Whilst they hold a weekly meeting where cases from across the country can be discussed, there is also flexibility to arrange emergency meetings in recognition of the hyperacute nature of HLH. Despite this, a clear ‘parent’ team must be responsible for coordinating care.

